# Circular RNA identified from *Peg3* and *Igf2r*

**DOI:** 10.1371/journal.pone.0203850

**Published:** 2018-09-14

**Authors:** Bambarendage P. U. Perera, Subash Ghimire, Joomyeong Kim

**Affiliations:** Department of Biological Sciences, Louisiana State University, Baton Rouge, Louisiana, United States of America; Montana State University Bozeman, UNITED STATES

## Abstract

Circular RNA is a newly discovered class of non-coding RNA generated through the back-splicing of linear pre-mRNA. In the current study, we characterized two circular RNAs that had been identified through NGS-based 5’RACE experiments. According to the results, the *Peg3* locus contains a 214-nucleotide-long circular RNA, circPeg3, that is detected in low abundance from the neonatal brain, lung and ovary. In contrast, the *Igf2r* locus contains a group of highly abundant circular RNAs, circIgf2r, showing multiple forms with various exon combinations. In both cases, the expression patterns of circPeg3 and circIgf2r among individual tissues are quite different from their linear mRNA counterparts. This suggests potential unique roles played by the identified circular RNAs. Overall, this study reports the identification of novel circular RNAs specific to mammalian imprinted loci, suggesting that circular RNAs are likely involved in the function and regulation of imprinted genes.

## Introduction

Circular RNA is a newly discovered class of non-coding RNAs that are produced through the back-splicing of linear pre-mRNA [1, 2]. In back-splicing, the splicing acceptor site of an upstream exon is joined to the splicing donor site of its downstream exon. In eukaryotic genes, the exons localized in the 5’-side tend to be included as circular RNA more frequently than those in the 3’-side. In particular, the 2nd exon is the most frequent exon that is included as part of circular RNA [3, 4]. Circular RNA is very stable due to its unusual circular structure, which lacks the 5’ cap and 3’ Poly-A tails [3]. As a consequence, circular RNA detection has been elusive until recent advancements in high-throughput sequencing, although some circular RNAs are quite ubiquitous and abundant *in vivo* [1, 2, 5]. Since its initial discovery from RNA viruses, recent studies indicate that circular RNAs are well conserved across mammals ranging from mice, porcine, to humans [1, 2]. In terms of physiological roles, circular RNAs are closely associated with various diseases, particularly in cancers, Alzheimer’s, neurological diseases, and diabetes. Thus, many circular RNAs have been recently recognized as biomarkers with potential for clinical diagnosis and therapeutic targets [6–8]. In some cases, circular RNA has been shown to function as a molecular sponge to remove microRNAs as means of regulating transcription [9, 10]. Besides these known functions, circular RNAs are predicted to be involved in many biological processes, including brain development, cellular stress, and aging [11, 12]. Nevertheless, the detailed mechanisms by which circular RNAs are involved in these processes are currently unknown.

In mammalian genomes, a subset of genes are expressed only from one allele due to an epigenetic mechanism termed genomic imprinting, by which one allele is usually repressed by DNA methylation and histone modifications [13, 14]. Imprinted genes tend to be clustered in specific regions of chromosomes, forming imprinted domains. The imprinting (mono-allelic expression) of several genes in a given domain is controlled through small genomic regions, termed Imprinting Control Regions [13, 14]. ICRs obtain allele-specific DNA methylation during gametogenesis, which is then maintained throughout the lifetime after fertilization [13, 14]. Many *cis*-regulatory elements are involved in the imprinting control of a given domain. In particular, alternative promoters located upstream of ICRs are known to be involved in establishing gametic DNA methylation on ICRs [15, 16]. To identify these alternative promoters for the *Peg3* domain, we previously performed several sets of Next Generation Sequencing (NGS)-based Rapid Amplification of cDNA Ends (RACE) experiments [17, 18]. Indeed, one of the identified alternative promoters, termed U1, is involved in establishing DNA methylation on the ICR of the *Peg3* domain [19, 20].

While analyzing the sequence data from the 5’RACE experiments, we serendipitously identified unusual circularized RNA transcripts that had been derived from several imprinted genes. In the current study, we have further characterized these potential circular RNAs with series of RT-PCR experiments. According to the results, two circular RNA, circPeg3 and circIgf2r, identified from *Peg3* and *Igf2r* imprinted loci, respectively, appeared to be genuine *in vivo* transcripts. Also, the expression patterns of these circular RNAs were quite distinct from their linear mRNA counterparts, suggesting potential unique roles by circular RNAs in genomic imprinting.

## Results

### Circular RNA identified from 5’RACE experiments

**Fig 1:**
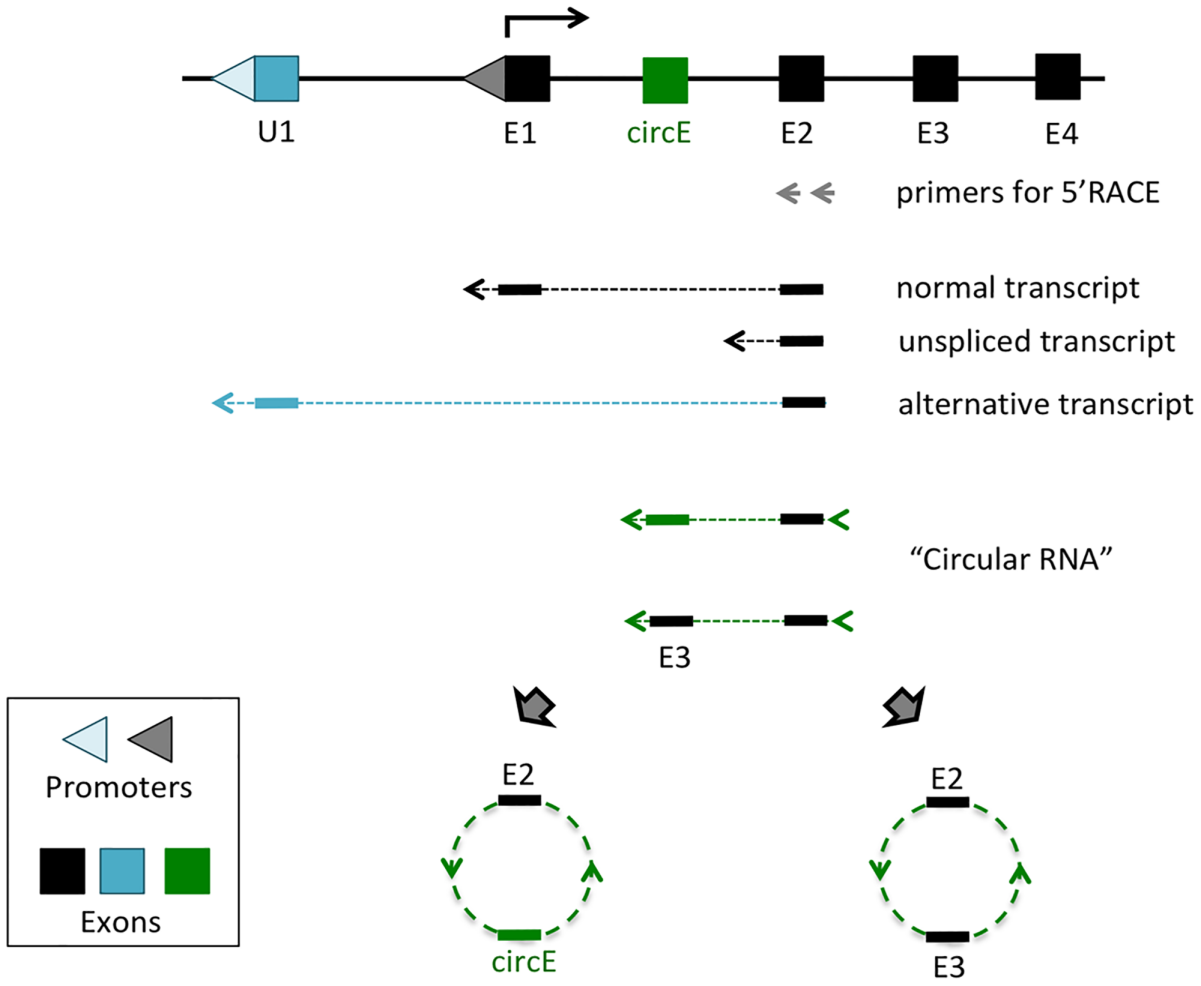
Circular RNA identified from 5’RACE experiments. Schematic representation of 5’RACE experiment. Total RNA isolated from tissues were first reverse-transcribed with the gene-specific primers that are derived from the 2nd exon of individual genes (grey arrows). The subsequent cDNA were further modified with G-tailing (arrowhead). These cDNA were amplified and used for NGS runs. Inspection of the sequence reads identified several different types of transcripts: normal transcripts with a proper joining of two exons (E1, E2), unspliced transcripts, and alternative transcripts driven by upstream alternative promoters/exons (U1). Detailed examination of the sequence reads also revealed the presence of circular RNAs: E2 + circE and E2 + E3.

Several sets of Next Generation Sequencing (NGS)-based Rapid Amplification of cDNA Ends (RACE) experiments were performed as part of ongoing efforts to identify upstream alternative promoters for several imprinted genes (Fig 1). For this series of experiments, total RNA was isolated from various mouse tissues, including hypothalamus, neonatal brain, ovary and testis. The isolated total RNA was first reverse-transcribed with the gene-specific primers that had been derived from the 2nd exons of imprinted genes, including *Snrpn*, *Zac1*, *Gtl2*, *Dlk1*, *Igf2r*, *Peg3*, and non-imprinted *Myc*, which served as a control. These cDNA were further processed with G-tailing followed by nested PCR amplification for NGS runs. We obtained total 2.5 million reads for this set of genes (S1 File). Initial inspection indicated that the majority of reads were derived from the three categories of transcripts: normal spliced transcripts with Exon1 (E1) and Exon2 (E2), unspliced transcripts and alternative transcripts starting from upstream alternative exons/promoters (U1) (Fig 1). Detailed inspection also revealed a fraction of raw reads with unusual exon combinations. The sequences from the 2nd exons of several genes were connected to the sequences of the 2nd exon itself, but through the 3’-side, or its downstream exons, forming potential circular RNAs via back-splicing events. The genes with these potential circular RNAs include *Peg3*, *Dlk1*, and *Igf2r*. In the case of *Peg3* and *Dlk1*, the circularized transcripts also contain previously unknown small exons that are derived from the 1st intron of each gene (S2 File). Interestingly, these exons are not included as part of the linear mRNA, but unique to the circular RNAs, thus named circular RNA-specific exons (circE). In the case of *Igf2r*, multiple forms of circular RNAs were detected, showing various exon combinations involving Exon 2 through 12. In terms of abundance, the sequence reads corresponding to the two circular RNAs, termed circPeg3 and circDlk1, accounted for less than 1% of the total number of the raw reads. On the other hand, the circular RNAs from the *Igf2r* locus, termed circIgf2r, accounted for about 20 to 50% of the total reads derived from each of the four tissue libraries, suggesting that circIgf2r may be a predominant group of transcripts *in vivo*.

### Circular RNA from the *Peg3* locus

**Fig 2:**
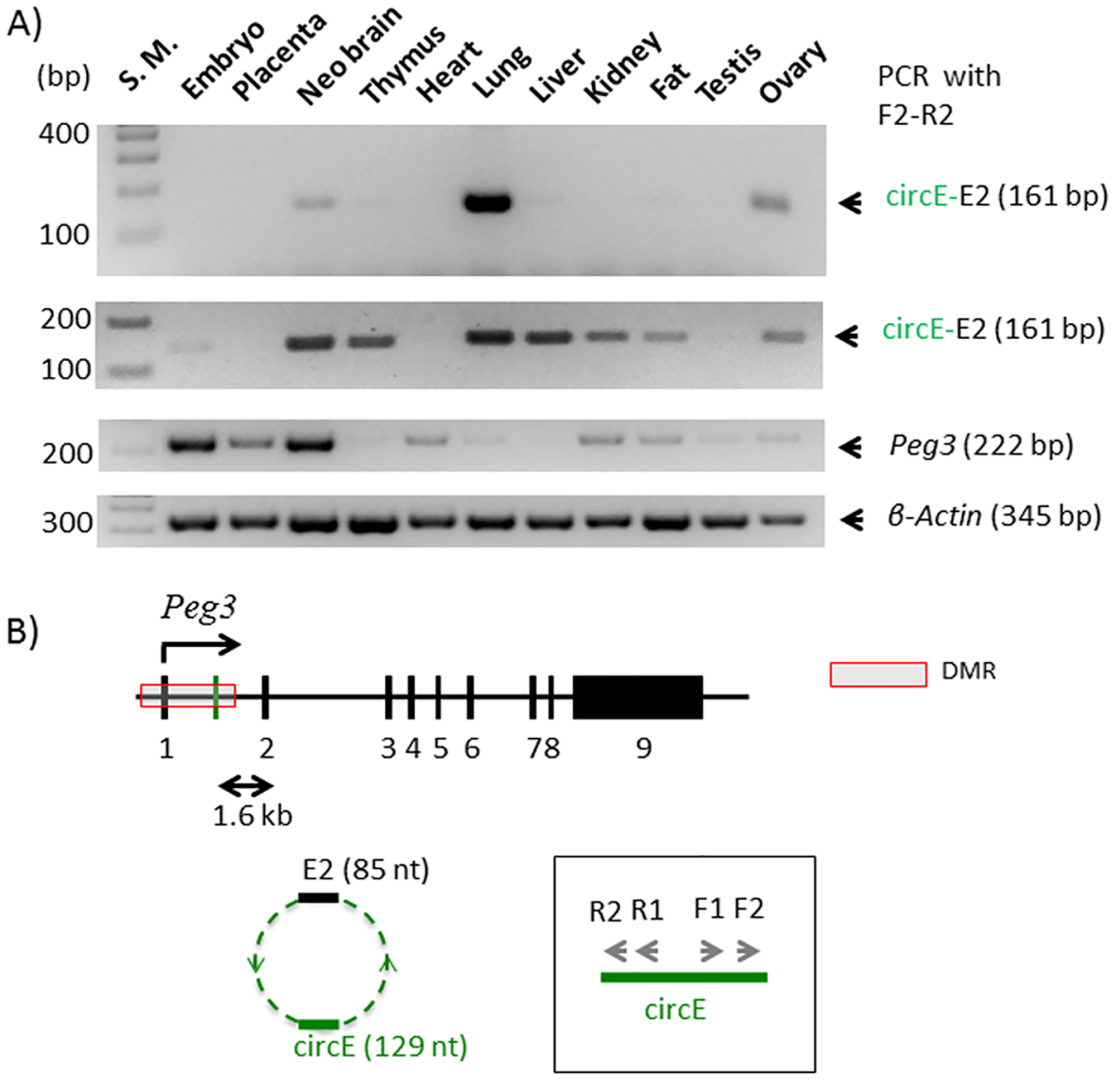
Circular RNA from the *Peg3* locus, circPeg3. (**A**) The two gel images on top represents the results of a nested PCR amplifying circPeg3 from two independent sets of cDNA that have been derived from the total RNA of various mouse tissues; the third image on middle shows the expression profile of *Peg*3; and the fourth image on bottom indicates the relative amount of cDNA between different tissues based on the expression levels of β-actin. (**B**) Schematic representation of the exon structure of the mouse *Peg3* locus. The black vertical lines indicate individual exons and the green vertical line indicates the circE exon. The arrows within the box indicate the two sets of primers that have been used for a nested PCR scheme to amplify circPeg3.

According to the results, the predicted circular RNA from the *Peg3* locus, circPeg3, is 214 nucleotide (nt) long, and made of two exons: the 2nd exon of *Peg3* and the circE exon, with 85 and 129 nt in length, respectively (Fig 2). The circE exon is localized 1.6-kb upstream of the 2nd exon of *Peg3* (chr7: 6,680,855–6,680,983 in mm9). This genomic region is still part of the 4-kb Peg3-DMR (Differentially Methylated Region), an Imprinting Control Region for the *Peg3* imprinted domain. This DMR is also known to have many YY1 binding sites. In fact, the 129-nt-long circE exon also contains one YY1 binding site (S2 File).

The predicted circPeg3 was further confirmed through a nested RT-PCR scheme involving two sets of primers with divergent orientation, which were derived from the circE exon (R1/F1 and R2/F2 in Fig 2B). For this test, we used a panel of cDNA that had been prepared from the total RNA isolated from various mouse tissues (Fig 2A). As expected, high levels of *Peg3* expression were detected from 14.5-dpc (days postcoitum) embryo and placenta as well as neonatal brain [21–23]. Medium to low levels of the expression were also detected from the remaining tissues (the third panel in Fig 2A). In contrast, low levels of circPeg3 were detected from neonatal brain, lung, and ovary after two rounds of nested PCR, indicating that the expression levels of circPeg3 are overall very low in the observed tissues. This is also consistent with the small number of the sequence reads detected from the initial NGS runs corresponding to circPeg3, which accounted for less than 1% of the total reads. We repeated this series of RT-PCR with another set of biological replicates, successfully detecting low levels of circPeg3 from various adult tissues, including neonatal brain, thymus, lung, liver, kidney, fat, and ovary (the second panel in Fig 2A). It is salient to note that there was no correlation between the expression levels of *Peg3* compared to the detection of circPeg3 among the tested tissues. Overall, this series of RT-PCR analyses confirmed the presence of low-abundant circPeg3 in the various tissues *in vivo*.

### Formation of circPeg3 in various mutant alleles

**Fig 3:**
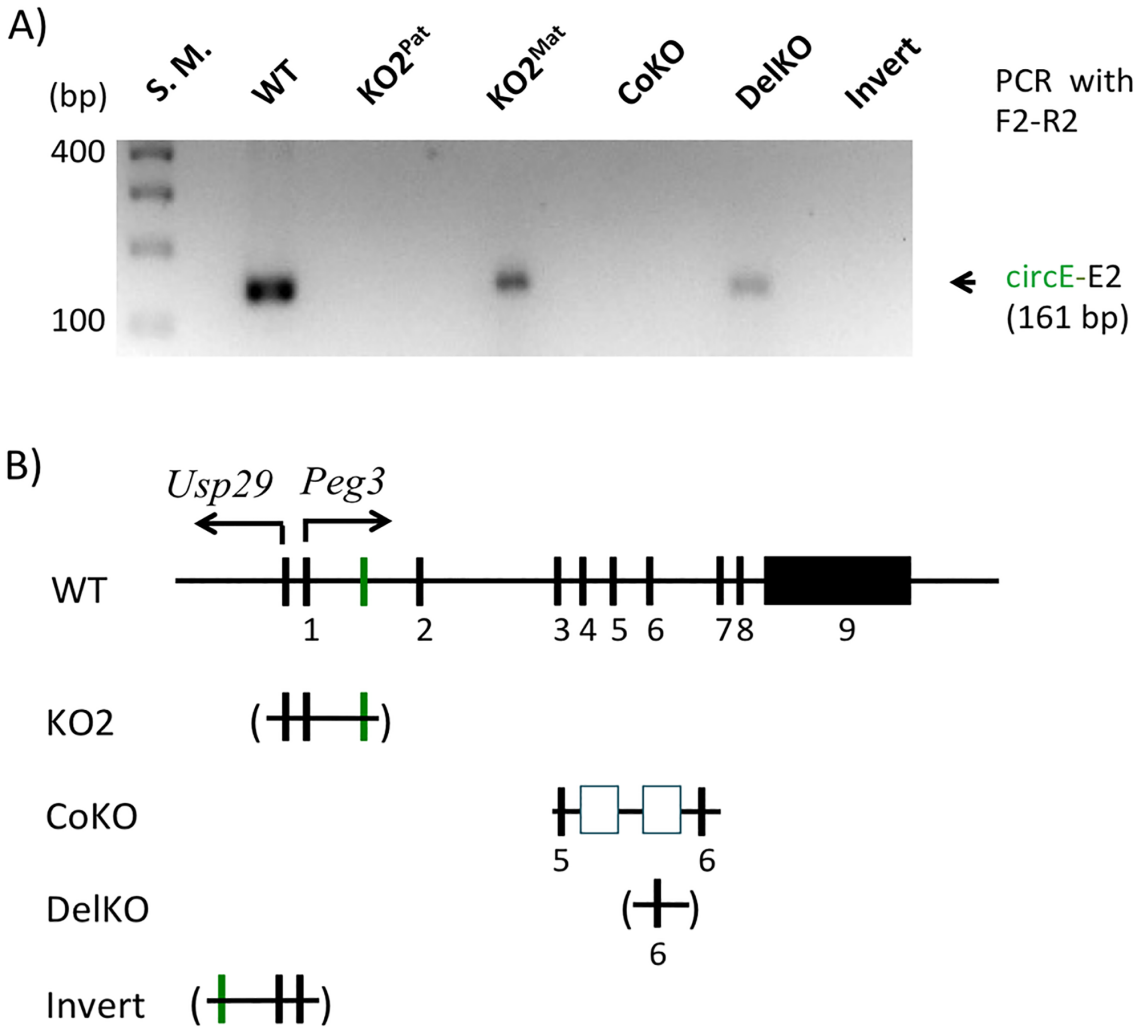
Formation of circPeg3 in various mutant alleles. (**A**) The gel image represents the results of a nested PCR amplifying circPeg3 from a set of cDNA that have been derived from various mutant alleles. (**B**) Schematic representation of the mutant alleles used for the current study. The exons are indicated with black vertical lines, while the circE exon is indicated with a green vertical line. The 4-kb genomic region corresponding to the Peg3-DMR is deleted in the KO2 allele, which is indicated with a parenthesis. In the CoKO allele, a 7-kb expression cassette containing the β-galactosidase and neomycin resistance genes with Poly-A tails is inserted into the 5th intron, which is indicated with two open boxes. In the DelKO allele, the 6th exon is deleted as indicated with a parenthesis. In the Invert allele, the 4-kb Peg3-DMR is inverted relative to the orientation of the surrounding genomic regions.

We further tested the formation of circPeg3 in several mutant alleles targeting the *Peg3* locus (Fig 3). The 4-kb genomic interval encompassing the bidirectional promoter for *Peg3* and *Usp29* has been hypothesized to be an ICR for this imprinted domain, thus this region has been targeted multiple times through mouse knockout experiments. One of the mutant alleles, termed KO2, contains a deletion of this 4-kb genomic region [21]. Since the circE exon is also localized within the same deleted region, we tested the formation of circPeg3 in the mutant animals with either paternal or maternal transmission of the KO2 allele (lane 3 and 4 in Fig 3). The results indicated that circPeg3 was detected in the mutant with the maternal transmission but not with the paternal transmission of the KO2 allele, confirming that circPeg3 originates from the paternal allele of *Peg3*. This also agrees with the paternal-specific expression of the linear mRNA counterpart of *Peg3* [22, 24, 25]. We further tested the formation of circPeg3 in additional mutant animals. The CoKO allele contains a 7-kb insertion of the expression cassette containing the β-galactosidase and neomycin resistance genes at the 5th intron of the *Peg3* locus (Fig 3B). In this mutant allele, the transcription of *Peg3* becomes truncated due to the two Poly-A signals included in this expression cassette [26, 27]. circPeg3 was not detected in the animals with paternal transmission of the CoKO allele (lane 5), thus suggesting that the formation of circPeg3 may require transcription of the entire length of the *Peg3* locus. The DelKO allele contains a deletion of the 1-kb genomic interval encompassing Exon 6 of *Peg3*, yet the transcription and subsequent splicing of this mutant allele has been shown to be normal according to the previous studies [27]. circPeg3 was also detected in the animals with paternal transmission of DelKO (lane 6), confirming no obvious effects on the formation of circPeg3 by the deletion of Exon 6. Finally, the 4-kb genomic interval of the Peg3-DMR was inverted in one mutant allele, termed Invert [28]. In this mutant allele, the promoters and 1st exons are exchanged between *Peg3* and *Usp29*. The circE exon is also part of the inverted region, thus localized in the direction of *Usp29* in this mutant allele. According to the previous study, the transcription and splicing of both genes normally occur in this mutant, but as fusion transcripts [28]. The 1st exon of *Peg3* is now connected to the 2nd exon of *Usp29*, while the 1st exon of *Usp29* is connected to the 2nd exon of *Peg3*. Nevertheless, circPeg3 was not detectable in the animals with paternal transmission of the inverted allele (lane 7 in Fig 3), thus suggesting that the formation of circPeg3 may require both the circE and 2nd exons of *Peg3*. Overall, this series of analyses concluded that circPeg3 originates from the paternal allele, and further that the formation of circPeg3 requires the transcription of the entire *Peg3* locus and also the 2nd exon of *Peg3*.

### Circular RNA from the *Igf2r* locus

**Fig 4:**
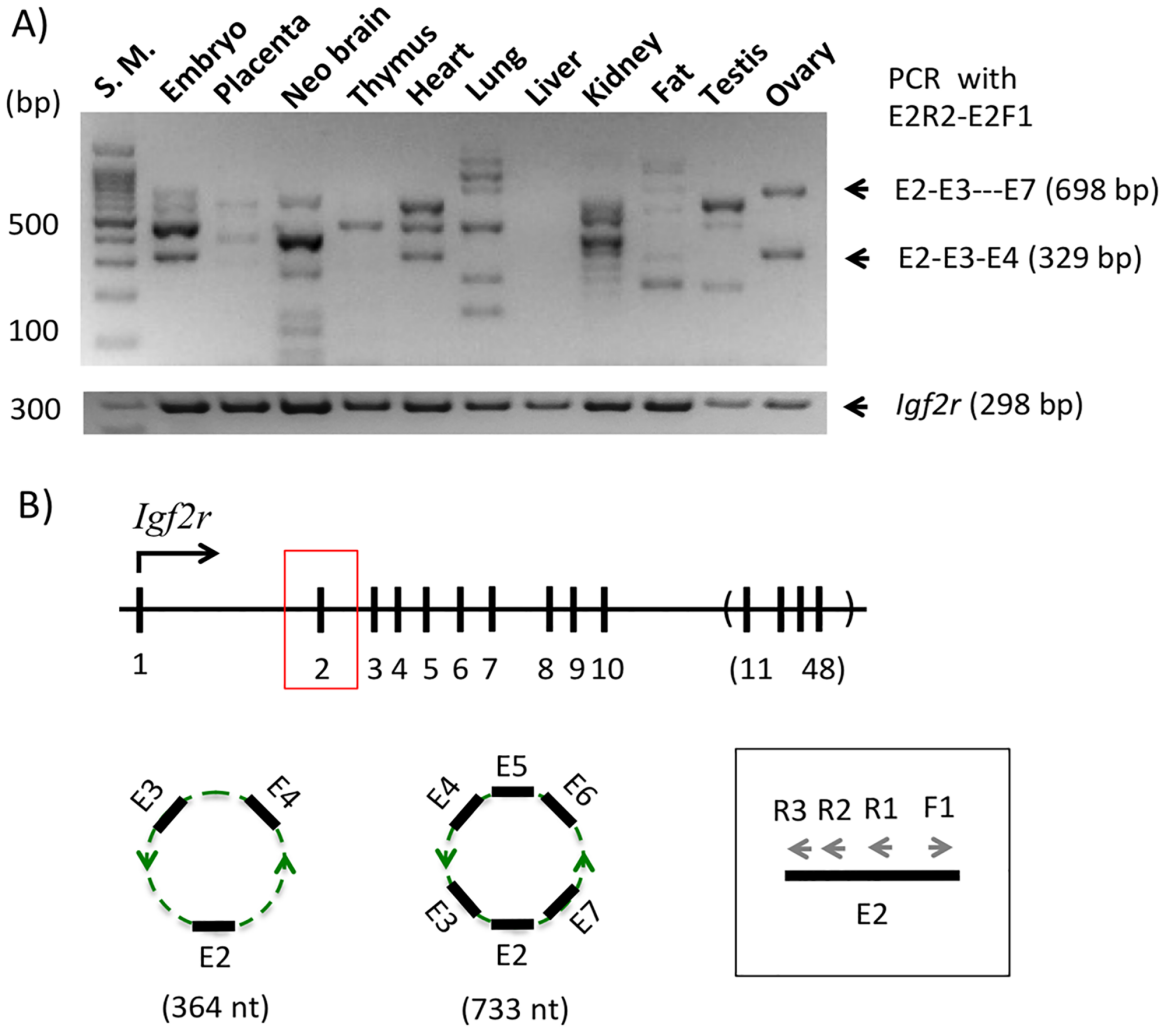
Circular RNA from the *Igf2r* locus, circIgf2r. (**A**) The gel image on top represents the results of the first PCR amplifying circIgf2r from a set of cDNA that have been derived from the total RNA of various mouse tissues and the image on bottom shows the expression profile of *Igf2r*. (**B**) Schematic representation of the exon structure of the mouse *Igf2r* locus. The black vertical lines indicate individual exons and the vertical lines within a parenthesis indicate the simplified version of the remaining exons, Exon 11 through 48. The arrows within the box indicate the primers that have been used for a nested PCR scheme to amplify circIgf2r.

**Fig 5:**
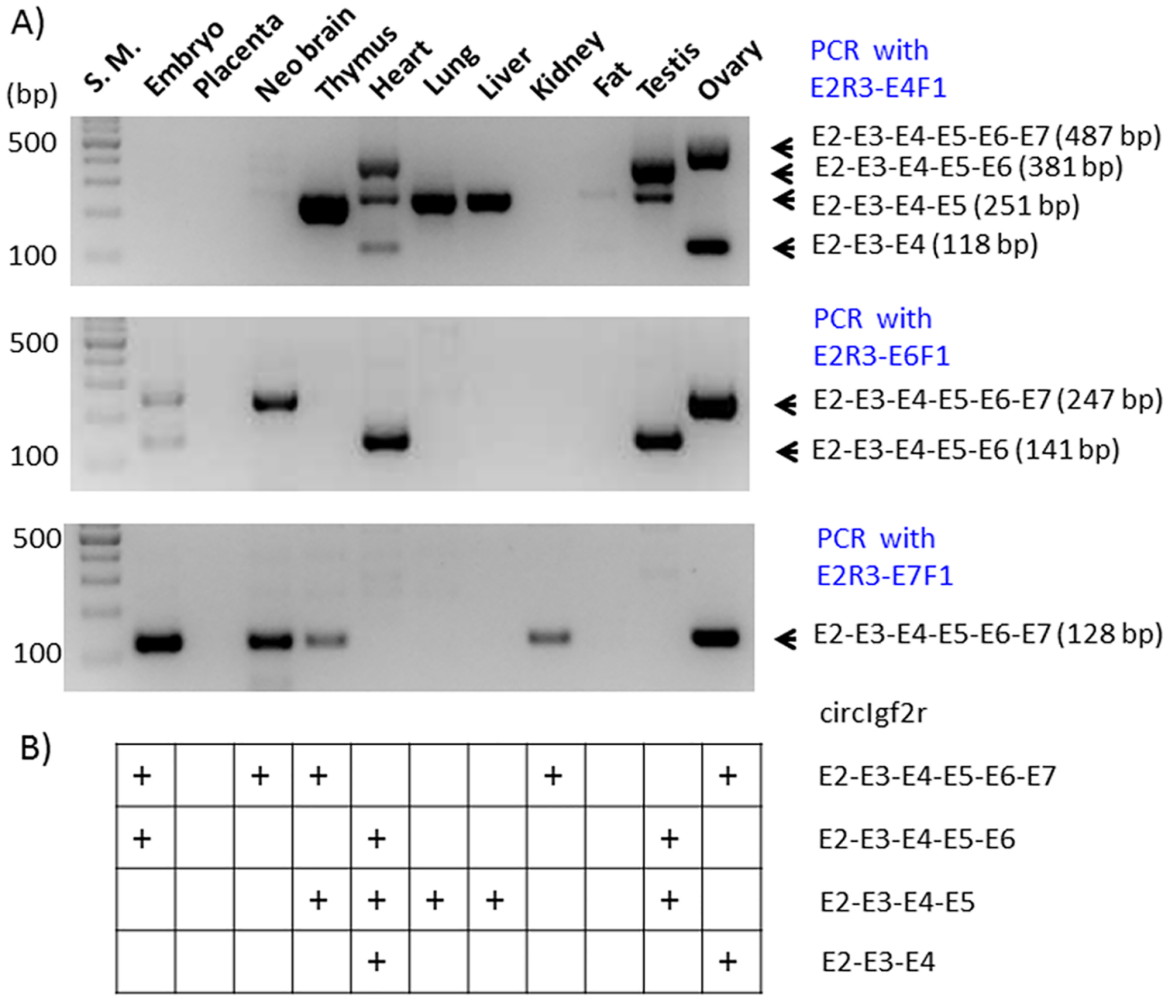
Multiple forms of circIgf2r with various exon combinations. (**A**) The gel image on top represents the result of a nested PCR with the primer combination of E2R3-E4F1; the image on middle represents those with the primer combination of E2R3-E6F1; and the image on bottom represents those with the primer combination of E2R3-E7F1. (**B**) The table summarizes the detection of each circular form of circIgf2r in a given tissue, which is indicated by "+".

The potential circular RNA from the *Igf2r* locus, termed circIgf2r, was also characterized in a similar way as described above (Fig 4). According to the results from the NGS runs, multiple forms of circIgf2r likely exist with various exon combinations. Also, circIgf2r is likely a group of major transcripts based on the sequencing results that the sequence reads corresponding to circIgf2r accounted for 20 to 50% of the total reads in the given library (S1 File). In this case, a slightly modified nested PCR scheme was employed to detect multiple forms of circIgf2r. First, an initial RT-PCR was performed with a set of divergent primers that were derived from the 2nd exon of *Igf2r*. As demonstrated for circPeg3 (Fig 2), the same cDNA panel was also used for circIgf2r detection. This initial PCR amplified a large number of PCR products that were readily detectable from multiple tissues (Fig 4A). Yet, the sizes of these PCR products were discrete but not contiguous, showing an about 100-bp difference between two adjacent PCR products. The *Igf2r* locus is made of 48 exons with each exon averaging 100 bp in length, particularly from Exon 2 through 10 (Fig 4B). Thus, this may be an indication that this initial PCR might have amplified multiple forms of circIgf2r with different exon combinations. To test this possibility, we performed the 2nd nested PCR with a slightly modified scheme. In this scheme, the first primer targeted the 2nd exon of *Igf2r* in the reverse direction, E2R3, but a set of the second primers were designed from individual exons in the forward direction. According to the results from the NGS runs, Exon 2 was often connected to Exon 4, 6, 7 and 10. Thus, a set of four primers, including E4F1, E6F1, E7F1, and E10F1, were designed and used individually with the E2R3 primer to detect the corresponding circular RNA (Fig 5). PCR with the three combinations of primers except the E2R3-E10F1 combination successfully amplified the target products based on their expected sizes, ranging from 118 to 141 bp in length. The shortest form of circIgf2r, E2-E3-E4, was detected from heart and ovary (top panel), whereas the longest form, E2-E3-E4-E5-E6-E7, was detected from 14.5-dpc embryos, neonatal brain, thymus, kidney, and ovary (bottom panel). This series of PCR also detected another form, E2-E3-E4-E5, in several tissues, although this particular form had not been previously observed from the NGS runs. The two forms, E2-through-E5 and E2-through-E7, seemed to be detected more frequently than the other two, E2-through-E4 and E2-through-E6, in the tissues examined so far **(**Fig 5B). This series of analyses were repeated with another set of biological replicates (S3 File). Ten out of the 15 detected cases were reproducible between these two biological replicates. Overall, this series of analyses confirmed that circIgf2r exists as multiple forms with various exon combinations *in vivo*.

## Discussion

In the current study, two circular RNAs, circPeg3 and circIgf2r, identified from the *Peg3* and *Igf2r* imprinted loci were further characterized by series of RT-PCR analyses. circPeg3 is a relatively low abundant transcript detected in various mouse tissues. In contrast, circIgf2r represents a group of high abundant transcripts displaying multiple forms with various exon combinations. According to the results, the expression patterns of these two circular RNAs among individual tissues were quite different from the expression patterns of the linear mRNA counterparts, suggesting potential unique roles played by these two circular RNAs.

The experimental strategy used for the current study, NGS-based 5’RACE experiments, appeared to be quite successful for identifying circular RNA (Fig 1). Three potential circular RNAs have been identified from the seven genes tested so far, yet two of these turned out to be genuine *in vivo* transcripts according to the results from RT-PCR analyses (Figs 3 and 5). This success may be contributed by several factors. First, using unpurified total RNA rather than purified mRNA may have increased the chance of identifying circular RNA, given the fact that circular RNAs lack Poly-A tails [3]. This is also likely one of the main reasons why we previously failed to detect highly abundant circular RNAs despite numerous trials of RNA-seq experiments. Second, reverse transcription starting from the 2nd exons of individual genes may have also increased the odds of identifying circular RNA, since the 2nd exons of individual genes are most frequently involved in the formation of circular RNAs [3, 4]. Finally, recent advancements of NGS-based sequencing approaches have definitely increased the chances of finding low abundant circular RNA, which was demonstrated through the successful identification of circPeg3. Overall, although serendipitous but not planned, the current approach turns out to be an effective approach for identifying circular RNA.

The expression patterns of two circular RNAs, circPeg3 and circIgf2r, are quite different from those of the linear mRNA counterparts among the tested tissues. In the case of the *Peg3* locus, circPeg3 was detected in neonatal brain, lung, and ovary, although the expression levels of the linear mRNA counterpart, *Peg3* transcript, were very low in these tissues, especially in ovary and lung (Fig 2). This is also the case for the *Igf2r* locus. In this case, the expression of each form of circIgf2r appeared to be unique to each tissue. For instance, the frequent forms detected in the ovary were the E2-through-E4 and E2-through-E7 exon combinations, whereas the frequent form in lung, kidney, and testis was the E2-through-E5 combination (Fig 5). It is interesting to note that none of the tissues examined so far have all four forms. Thus, it is unlikely that these various forms of circIgf2r represent the erroneous byproducts that are generated during the splicing process of the linear pre-mRNA. In a similar context, it is also relevant to note that the small exon specific to circular RNA, circE in the case of circPeg3, contains a DNA-binding site for YY1. Since YY1 binding sites are quite ubiquitous in the mammalian genomes [29], it is reasonable to predict that some of the small RNAs, such as miRNA or piwiRNA, may also contain YY1 binding sites. In this case, this DNA-binding site within circPeg3 could function as a bait to attract these miRNAs, thus rendering circPeg3 as a potential miRNA sponge [9, 10].

It is currently unknown whether a circular RNA plays similar functions as its corresponding host genes. Nevertheless, it is relevant to note that both imprinted genes, *Peg3* and *Igf2r*, play significant roles in mammalian reproduction [1, 2]. Also, *Peg3* is a well-known tumor suppressor, and its promoter region is usually hypermethylated in the patients of ovarian and breast cancers [25]. The expression levels of *Peg3* tend to be very low in dividing cells, such as stem cells, whereas its expression levels tend to be very high in differentiated cells, such as muscle and neuron cells. These patterns were also demonstrated in the results of RT-PCR (the third panel in Fig 2A), showing high expression levels in neonatal brains versus low expression levels in thymus, lung and liver. Interestingly, the detection of circPeg3 was more obvious and consistent in the tissues with low expression levels of *Peg3*. Thus, this may be an indication that circPeg3 might play opposing roles compared to *Peg3*, promoting cell division. If this is the case, characterizing the precise roles of circPeg3 should be of great interest in the near future. In conclusion, the two circular RNAs identified from the mouse *Peg3* and *Igf2r* loci are genuine *in vivo* transcripts.

## Materials and methods

### Ethics statement

All the experiments related to mice were performed in accordance with National Institutes of Health guidelines for care and use of animals, and also approved by the Louisiana State University Institutional Animal Care and Use Committee (IACUC), protocol #16–060.

### Mouse breeding

In the current study, we used the following mutant strains: *Peg3*^*CoKO/+*^, *Peg3*^*KO2/+*^, *Peg3*^*DelKO/+*^, *Peg3*^*Inv/+*^ [26–29]. For genotyping, genomic DNA was isolated from either clipped ears or tail snips by incubating the tissues overnight at 55°C in the lysis buffer (0.1 M Tris-Cl, pH 8.8, 5 mM EDTA, pH 8.0, 0.2% SDS, 0.2 M NaCl, 20 μg/ml Proteinase K). The isolated DNA was subsequently used for genotyping. The sex of the pups was determined through PCR using the following primer set: mSry-F (5’-GTCCCGTGGTGAGAGGCACAAG-3’) and mSry-R (5’-GCAGCTCTACTCCAGTCTTGCC-3’). The information regarding individual primer sequences for each mutant allele is available through previous studies [25–28].

### NGS-based 5’RACE experiments

Tissues were collected from hypothalamus, testis, liver, heart, and kidney from an adult male mouse (WT); ovaries were collected from an adult female (WT); a whole head was used from a one-day-old neonate (WT). The tissues were subject to total RNA isolation using the Trizol RNA isolation kit (Invitrogen). The resulting total RNA (2.5–5 μg) was mixed with gene-specific primers corresponding to the second exons for *Peg3*, *Gtl2*, *Dlk1*, *Igf2r*, *Snrpn*, *Zac1*, and *Myc* (S4 File), and reverse-transcribed using the M-MuLV reverse transcriptase (New England Biolabs, Cat. No. M0253S). The cDNA products were purified using phenol/chloroform extraction and ethanol precipitation. The 3′-ends of the purified cDNA was further modified by the tailing reaction using dGTP and terminal deoxynucleotidyl transferase according to the manufacturer’s protocol (New England Biolabs, Cat. No. M0315S). The tailed cDNA was amplified using two primers: the tail-long primer (5′ -GGTTGTGAGCTCTTCTAGATCCCCCCCCCCCCNN-3′) and internal gene-specific primers to check for quality (S4 File) [17, 18]. The amplified cDNA was re-amplified with a set of nested primers: the tail-out primer (5′-GGTTGTGAGCTCTTCTAGA-3′) and additional internal gene-specific primers to increase the possibility to detect low abundant transcripts. The PCR products were further purified, multiplexed, and sequenced according to a next generation sequencing (NGS) protocol [17, 18]. Additional RT-PCR reactions were also performed to monitor the quality of cDNA, and also the specificity of the tailing reaction *via* the gene-specific forward and reverse primers that are derived from the 1st and 2nd exons, respectively (S4 File). The first round of NGS-based 5’RACE included the hypothalamus, neonate head, ovary, and testis tissues to identify potential alternative promoters for *Snrpn*, *Zac1*, *Gtl2*, *Dlk1*, *Igf2r*, and *Myc*. The second round of NGS-based 5’RACE included the testis, ovary, heart, and kidney tissues to identify potential alternative promoters for *Peg3* (S1 File). The results from these NGS runs have been deposit to the SRA database (SRA Accession No. SRP156941).

### RT-PCR

Several sets of cDNA panel were generated using the total RNA isolated from various mouse tissues. In brief, the total RNA was isolated from each tissue using the Trizol RNA isolation kit (Invitrogen). The isolated total RNA (2.5–5 μg) was mixed with random hexamers, and subsequently reverse-transcribed using the M-MuLV reverse transcriptase (New England Biolabs, Cat. No. M0253S). The resulting cDNAs were used as templates for detecting circular RNAs and linear mRNA transcripts for each imprinted gene. Nested PCR schemes were used for the detection of circular RNA with the following PCR condition. The initial PCR for circular RNAs was performed in the following parameters: 35 cycles of 30 seconds at 95°C, 30 seconds at 60°C, 30 seconds at 72°C. The annealing temperature for circIgf2r was 60°C. One μl of the initial PCR products was diluted 10-fold with water, and these diluted PCR products were subsequently used as templates for the second PCR with the following parameters: 20 to 25 cycles of 30 seconds at 95°C, 30 seconds at 62°C, 30 seconds at 72°C. We also performed a set of control experiments with the AMV reverse transcriptase (New England Biolabs, Cat. No. M0277S) to ascertain that the RT-PCR products obtained through the M-MuLV reverse transcriptase are derived from genuine circular RNAs (S5 File). The information regarding the sequences of the primers used for these PCR are available (S2 and S4 Files).

## Supporting information

S1 File Summary of the results from NGS-based 5’RACE experiments. This file contains the number of raw sequence reads for each gene, and also the summary of the mapping results showing the number of circular RNA transcripts in each library.
(XLSX)

S2 File Sequences and exon structures of the identified circular RNAs. This file contains the information regarding the sequences and exon structures of the three circular RNAs, circPeg3, circDlk1, and circIgf2r.
(DOCX)

S3 File Multiple forms of circIgf2r with various exon combinations. This file contains the results derived from the nested PCR amplifying circIgf2r from the 2nd set of biological replicate.
(PPTX)

S4 File Primers used for NGS-based 5’RACE experiments. This file contains the sequences of all the oligonucleotides used for 5’RACE experiments.
(DOCX)

S5 File Control experiments for RT-PCR-based detection of circPeg3. This file contains a set of RT-PCR products derived from circPeg3 with two different reverse transcriptases, M-MuLV and AMV.
(PPTX)
