# Supplementary material for: Circular RNA identified from Peg3 and Igf2r
Source: PLoS One. 2018 Sep 14;13(9):e0203850. doi: 10.1371/journal.pone.0203850 (PMC6138396; doi:10.1371/journal.pone.0203850)
Supplement: S2 File — This file contains the information regarding the sequences and exon structures of the three circular RNAs, circPeg3, circDlk1, and circIgf2r. (DOCX) [file pone.0203850.s002.docx]

**Sequences and Exon structures of three circular RNAs**

*Two of the circRNAs predicted through NGS runs, except circDlk1, have been demonstrated to be *in vivo* transcripts, thus described in detail in the manuscript.

>circPeg3 (214 nt long)

CTGGACCAGCCACCTAGCTGTTGGAGGACCTAGTCTTCCTCTTGCCAGTTGTCTCCAAAAAGGATAGAAGATCAAGAAGGTAGGGCATCAGTCAGGATAATAACCCCTTCCTAGGGCTTGCCCCTTGCAAGACAGCTTAGCACTCAGGACCATAAATCCTCTCTCTAGCCTGCCTCTTGCAGCCAGCCAGTCCAGCTGTCAATCAAAATGGCAC

The 2nd exon of *Peg3* is marked with the yellow background, while a new exon specific for a circular RNA is marked with blue. The region with red corresponds to the YY1 binding site.

Primers used for the detection of circPeg3

>circPeg3-R1

AGAGAGGATTTATGGTCCTGAGT

>circPeg3-F1

GCCTGCCTCTTGCAGCCAGCCA

>circPeg3-R2

TAAGCTGTCTTGCAAGGGGCAAG

>circPeg3-F2

CAGCTGTCAATCAAAATGGCAC

Primers used for the detection of Peg3 (222 bp)

>Peg3-RT-1a

GGTTCAGTGTGGGTGCACTAGACT

>Peg3-RT-1b

GCTCACACCCAAGGGCTTGAGCG

>circDlk1 (118 nt long)

CTGCAGACATTGTCAGCCTCGCAGAATCCATACTGGGGGTCACAGGGTGGGTCGCATTCAGCCCTCCCACGTTTCTCCTACCCCACACTGGTCACATTAAAAGAACTGCGCAATAGCT

The 2nd exon of *Dlk1* is marked with the yellow background, while a new exon specific for a circular RNA is marked with blue.

Primers used for the detection of circDlk1

>circDlk1-F1

CACATTAAAAGAACTGCGCA

>circDlk1-R1

CAGTGTGGGGTAGGAGAAAC

>circDlk1-F2

ATTAAAAGAACTGCGCAATAG

>circDlk1-R2

TGGGGTAGGAGAAACGTGGGA

Primers used for the detection of Dlk1 (64 bp)

>mDlk1-RT-Ex1-F1

CTTTCGGCCACAGCACCTATG

>mDlk1-RT-Ex2-R2

CAGAATCCATACTGGGGGTCAC

>circIgf2r (E7-E6-E5-E4-E3-E2; 733 nt long)

CTCTCTCCGCTCAGATGGGCACACAAATGTTACTGTCACTGCAGGGCTGTGACCATTACAGAAGTCTGGCTTTTCTCCCTCTTCCTTCACATAAGTCAGAACGAGC

CTGTCCTTGCTCAACAGCTTCAGCCCCTCCTTGGGTCGGCCAACGTCAAATGCCTGGTTCCCCTTCAGCAGACAGGCAGCAGTGCCAGCAGGACAGACTCTCAGCTGTGTGCTGGGGTCCCGAAGGGAGT

CTATGTCTCTACACACGTTGATGAACAGAGACGTGTCAGGATCAGAATCATCTACCAGATAGCCACCATTTAGCTTGATCAGTGGGTTTAAATCATGCTTCTGTAACTTGTCATCAAATGCATAGCATGGTAC

CTCCTTATCAGCTTTAAATATGTCTTTCTTGCAGGCTGCAGTAGTCCTCCATTCAAAGTAATGCACACAGTCTGTGGCAGTTACAAATTCAGGAGTTCC

CAGGGTCTTCCCACACAGAAATGTAATGCTAGTCTGGATTCTGTGCTGTGAATCTGAAGGCTGGCAGCCCATAGTGGTGTTGAATTCCAGGAGTGATCTGGCTGAACTTCTCAAAAGTGAGTCAC

CCACTGAGCGGCAGTTCTCTGTCTTTAGGTCACACATACAGATAGCACTTGTAGGCCCACAGCTGGAAATACCCACATTTCCACAGACGTTGATCTTATAAACTGCATTGTTTTTGGAGTCAACAGCTTCCCATGTGTAA

Primers used for the detection of circIgf2r

>mIgf2r-Ex2-R2

CAGCTGGAAATACCCACATTTC

>mIgf2r-Ex2-F1

ACAGAGAACTGCCGCTCAGTGG

>mIgf2r-Ex2-R3

GGAGTCAACAGCTTCCCATGTG

>mIgf2r-Ex4-F1

ACTCCTGAATTTGTAACTGCCA

>mIgf2r-Ex6-F1

GACCCCAGCACACAGCTGAGAGT

>mIgf2r-Ex7-F1

GCTCGTTCTGACTTATGTGAAG

>mIgf2r-Ex10-F1

TCAGATGGAGACCTCACCCTCA

Primers used for the detection of Igf2r (298 bp)

>mIgf2r-F2

TCAGTATGCAAAGTTCCTGTGG

>mIgf2r-R2

GCTCATCTGTTACAGCACAACC
