# Supplementary material for: Circular RNA identified from Peg3 and Igf2r
Source: PLoS One. 2018 Sep 14;13(9):e0203850. doi: 10.1371/journal.pone.0203850 (PMC6138396; doi:10.1371/journal.pone.0203850)
Supplement: S3 File — This file contains the results derived from the nested PCR amplifying circIgf2r from the 2nd set of biological replicate. (PPTX) [file pone.0203850.s003.pptx]

## Slide 1
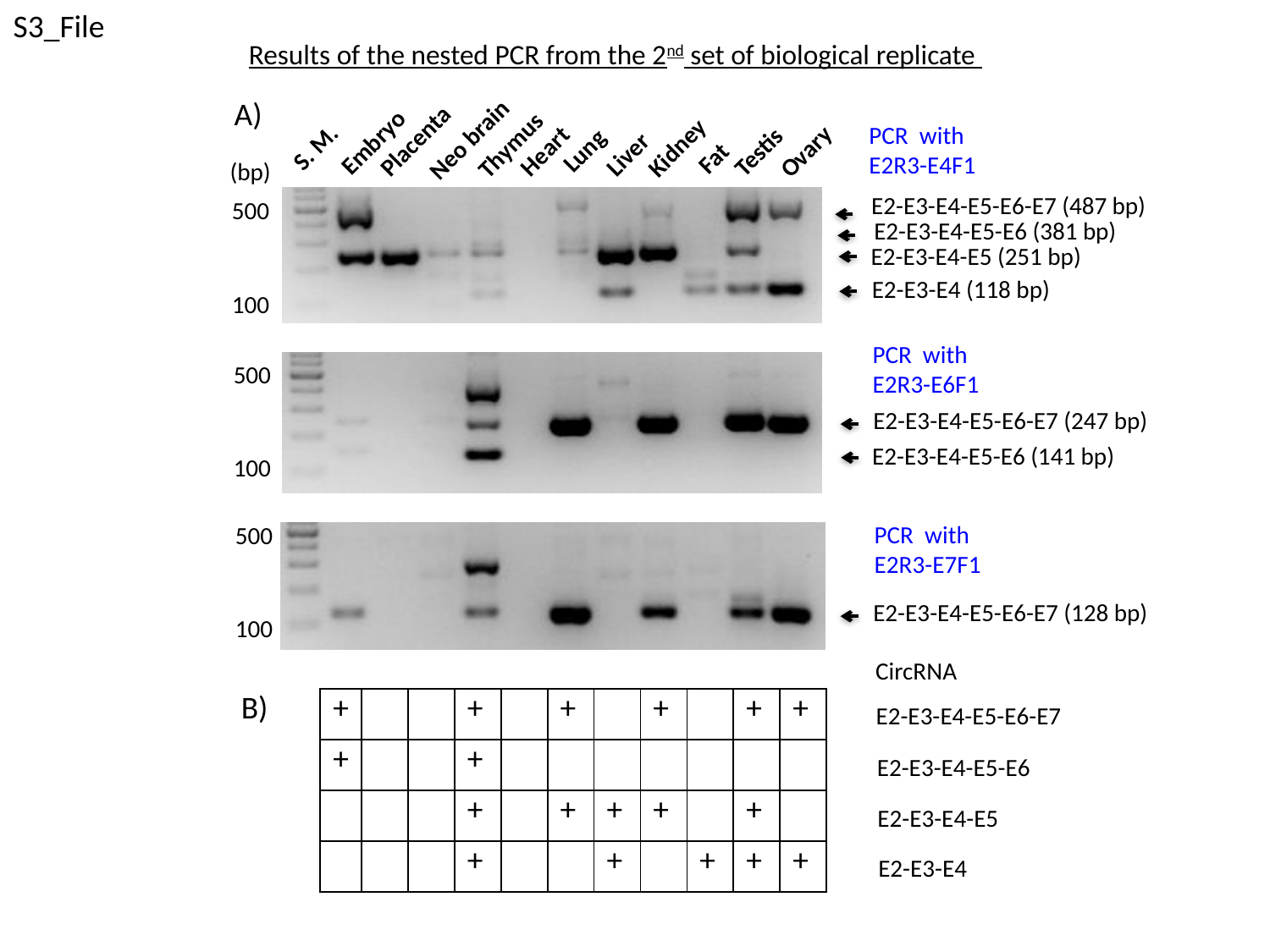

S3_File
Results of the nested PCR from the 2nd set of biological replicate
A)
PCR with
E2R3-E4F1
Neo brain
Placenta
Embryo
Thymus
S. M.
Kidney
Lung
Heart
Testis
Ovary
Liver
Fat
(bp)
E2-E3-E4-E5-E6-E7 (487 bp)
500
E2-E3-E4-E5-E6 (381 bp)
E2-E3-E4-E5 (251 bp)
E2-E3-E4 (118 bp)
100
PCR with
E2R3-E6F1
500
E2-E3-E4-E5-E6-E7 (247 bp)
E2-E3-E4-E5-E6 (141 bp)
100
PCR with
E2R3-E7F1
500
E2-E3-E4-E5-E6-E7 (128 bp)
100
CircRNA
B)
| + | | | + | | + | | + | | + | + |
| --- | --- | --- | --- | --- | --- | --- | --- | --- | --- | --- |
| + | | | + | | | | | | | |
| | | | + | | + | + | + | | + | |
| | | | + | | | + | | + | + | + |
E2-E3-E4-E5-E6-E7
E2-E3-E4-E5-E6
E2-E3-E4-E5
E2-E3-E4
