# Supplementary material for: Circular RNA identified from Peg3 and Igf2r
Source: PLoS One. 2018 Sep 14;13(9):e0203850. doi: 10.1371/journal.pone.0203850 (PMC6138396; doi:10.1371/journal.pone.0203850)
Supplement: S4 File — This file contains the sequences of all the oligonucleotides used for 5’RACE experiments. (DOCX) [file pone.0203850.s004.docx]

**Primers used for NGS-based 5'RACE experiments**

The first two primers (ExF1 and Ex2R1) were used for RT-PCR to check the quality of cDNA after the initial gene-specific reverse transcription reaction with the primer Ex2R1. After G-tailing reaction, the cDNA were amplified with nested PCRs: the first amplification with Ex2R2 and the tail long primer (GGTTGTGAGCTCTTCTAGATCCCCCCCCCCCCNN) followed by the second amplification with Ex2R3 and the tail out primer (GGTTGTGAGCTCTTCTAGA).

*Peg3*

>mPeg3-RT-Ex1-F1

GGTTCAGTGTGGGTGCACTAGACT

>mPeg3-RT-Ex2-R1

AGTCTTCCTCTTGCCAGTTGTC

>mPeg3-RT-Ex2-R2

TCCTCTTGCCAGTTGTCTCCAA

>mPeg3-RT-Ex2-R3

ATAGAAGATCAAGAAGGTAGGG

*Snrpn*

>mSnrpn-RT-Ex1-F1

AAACCTGAGCCATTGCGGCAAGAC

>mSnrpn-RT-Ex2-R1

CTTCGACGTTTGACCTGGACCT

>mSnrpn-RT-Ex2-R2

GTAGTTCTTCTCAAGTGTAAGC

*Zac1*

>Zac1-RT-Ex1-F1

GATCGGAGATCTGACGGCGAC

>Zac1-RT-1R

GAGAGTCTATCTTCGAGGAGAC

>Zac1-RT-Ex4-R1

TTAACTCCTCTGACTCCTATGC

>Zac1-RT-Ex4-R2

CCTCTGACTCCTATGCAAATAC

*Gtl2*

>mGtl2-RT-Ex1-F1

CGCCCACAGAAGAATCTCTTAC

>mGtl2-RT-Ex2-R1

TAGAGGTGCACAGCAGGTACTC

>mGtl2-RT-Ex2-R2

ACTGGTGTGAGCCGATGATGTC

>mGtl2-RT-Ex2-R3

ATGTCATCCCTGAAGAGAGAGC

*Dlk1*

>mDlk1-RT-Ex1-F1

CTTTCGGCCACAGCACCTATG

>mDlk1-RT-Ex2-R1

CTGCAGACATTGTCAGCCTCGC

>mDlk1-RT-Ex2-R2

CAGAATCCATACTGGGGGTCAC

>mDlk1-RT-Ex2-R3

TCACAGGGTGGGTCGCATTCAG

*Igf2r*

>mIgf2r-RT-Ex1-F1

CTCTTTCTCCCTCCAGCTCCCG

>mIgf2r-RT-Ex2-R1

GCGGCAGTTCTCTGTCTTTAGG

>mIgf2r-RT-Ex2-R2

CAGCTGGAAATACCCACATTTC

>mIgf2r-RT-Ex2-R3

GGAGTCAACAGCTTCCCATGTG

*Myc*

>mMyc-RT-Ex1-F1

CGCGATCAGCTCTCCTGAAAAG

>mMyc-RT-Ex2-R1

CTTCCAGATATCCTCACTGGGC

>mMyc-RT-Ex2-R2

CGCTCTGCTGTTGCTGGTGATAG

>mMyc-RT-Ex2-R3

GTTGGTGAAGTTCACGTTGAG
