# Supplementary material for: Circular RNA identified from Peg3 and Igf2r
Source: PLoS One. 2018 Sep 14;13(9):e0203850. doi: 10.1371/journal.pone.0203850 (PMC6138396; doi:10.1371/journal.pone.0203850)
Supplement: S5 File — This file contains a set of RT-PCR products derived from circPeg3 with two different reverse transcriptases, M-MuLV and AMV. (PPTX) [file pone.0203850.s005.pptx]

## Slide 1
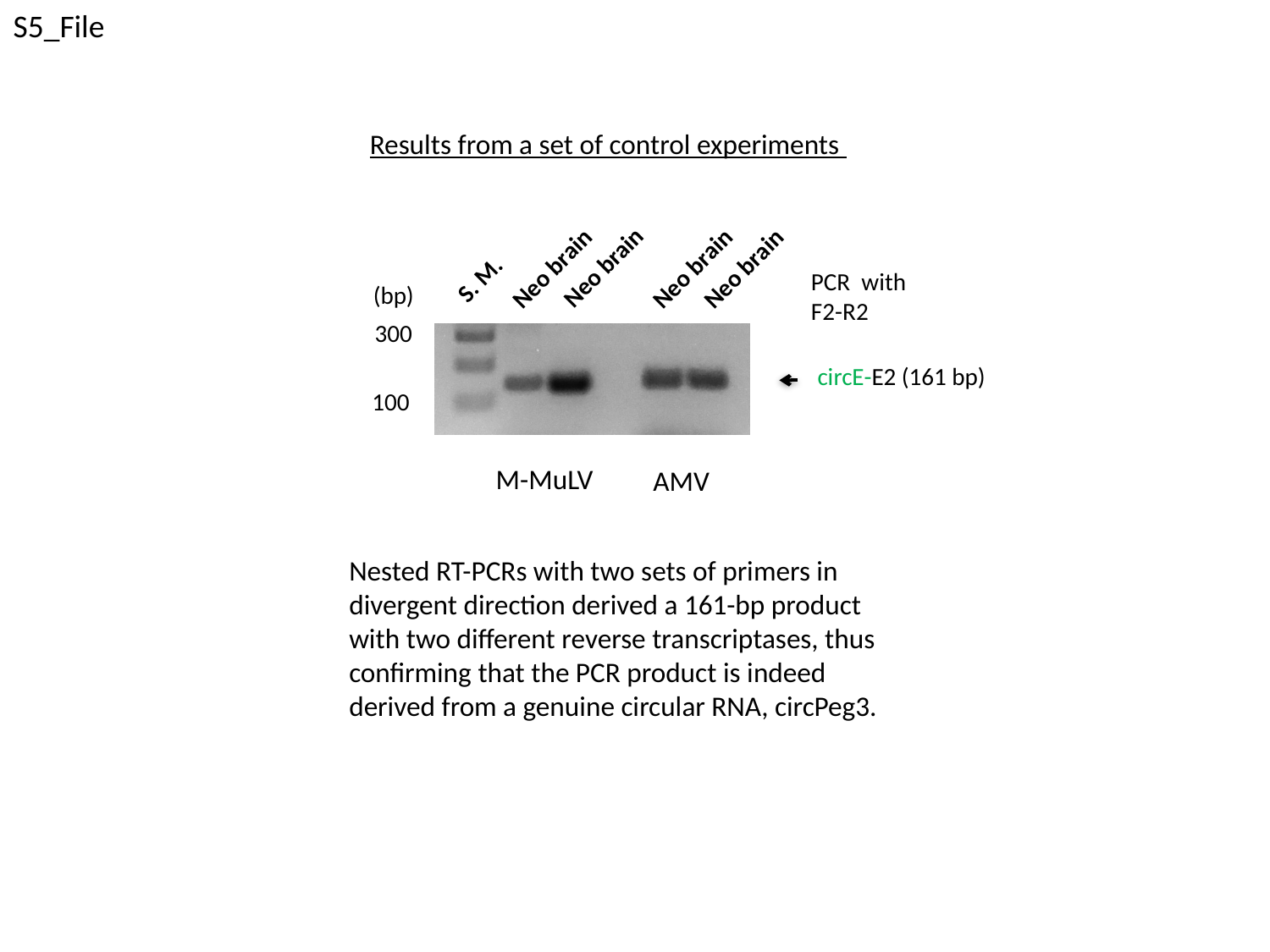

S5_File
Results from a set of control experiments
Neo brain
Neo brain
Neo brain
Neo brain
S. M.
PCR with
F2-R2
(bp)
300
circE-E2 (161 bp)
100
M-MuLV
AMV
Nested RT-PCRs with two sets of primers in divergent direction derived a 161-bp product with two different reverse transcriptases, thus confirming that the PCR product is indeed derived from a genuine circular RNA, circPeg3.
